# Our Journal

**Published:** 1854-02

**Authors:** 


Our Journal.—A medical friend, an honor to his profession, in a recent conversation, urged strongly the propriety and necessity of enlarging the Journal. We assured him that it was our determination so to do, as soon as the "life giving principle" is infused into it in the shape of a larger list of prepaying subscribers, and this is our settled purpose. Therefore those of our brethren who desire to see a larger Journal of their own, published with the promptness and regularity which characterizes it, will each on the receipt of this, consider himself a committee of one to procure an additional subscriber, and as soon as this is done, which *can* be done, our Journal will be increased in size. The same gentleman assured us that he would subscribe and pay for an additional number of copies, and distribute them among his medical friends, who he believed took no publication of the kind.

Another noble specimen of a medical man, writing from a county away off towwards sunset, enclosing the necessary nutrient element, requested to be enrolled as a "subscriber for life," or as long as the Journal continued to breathe. It gives us pleasure to announce a gradual increase of our subscription list, and therefore our friend may be sure of finding it for years to come, a familiar and we trust a profitable guest.

